# Assessing Fish Populations Through Length–Weight Relationships and Condition Factors in Three Lakes of Nigeria

**DOI:** 10.3390/biology14060612

**Published:** 2025-05-27

**Authors:** Olumide Temitope Julius, Francesco Zangaro, Roberto Massaro, Francesca Marcucci, Armando Cazzetta, Franca Sangiorgio, John Bunmi Olasunkanmi, Valeria Specchia, Oluwafemi Ojo Julius, Ilaria Rosati, Alberto Basset, Maurizio Pinna

**Affiliations:** 1 Department of Biological and Environmental Sciences and Technologies (DiSTeBA), University of Salento, Via Monteroni 165, 73100 Lecce, Italy; 2 Research Centre for Fisheries and Aquaculture of Aquatina di Frigole, DiSTeBA, University of Salento, Via Negri, 73100 Lecce, Italy; 3 National Biodiversity Future Center (NBFC), 90133 Palermo, Italy; 4 BioforIU Research Lab, DiSTeBA, University of Salento, Via Monteroni 165, 73100 Lecce, Italy; 5 Department of Fisheries and Aquaculture, Federal University Oye-Ekiti, Ikole 370105, Ekiti, Nigeria; 6 Department of Science Technology, The Federal Polytechnic, Ado-Ekiti 360231, Ekiti, Nigeria; 7 LifeWatch Service Centre, LifeWatch Italy, Via Monteroni 165, 73100 Lecce, Italy; 8 Research Institute on Terrestrial Ecosystems, National Research Council of Italy, 73100 Lecce, Italy; 9 Institute of Applied Sciences and Intelligent Systems “Edoardo Caianiello”, National Research Council of Italy, 73100 Lecce, Italy

**Keywords:** allometric growth, freshwater ecosystem, Nigerian lakes, water quality variables, growth coefficient

## Abstract

The fish populations of three lakes in Nigeria (Ureje, Egbe, and Ero) were assessed using tools such as the length–weight relationship and condition factor. The study lasted for 12 weeks (between July and October 2024) and used samples collected from fishermen’s catch. Species richness and relative abundances in the samples showed slight variations across the three study sites, with Ero having 945 individuals (9 species), Egbe having 733 individuals (8 species), and Ureje having 811 individuals (7 species). All specimens exhibited allometric growth with specimens from Ero Lake mostly showing positive allometric growth (b > 3), unlike their counterparts from other lakes, which varied between positive and negative allometric growth (b < 3). The condition factor across the three lakes ranged from 0.48 (*Parachanna obscura* in Ureje Lake) to 3.01 (*Tilapia zilli* in Ureje Lake). Even though there were no statistically significant differences (*p* > 0.05) between the condition factors of species across the three lakes, specimens from Ero lake generally appeared to be in better condition. In this study, no significant relationship was found between water quality variables and the growth coefficient (b) and condition factors. The study’s results provide sufficient information for planning and implementing conservation/management efforts in these lakes.

## Simple Summary

This study examined fish populations in three lakes in Nigeria (Ureje, Egbe, and Ero) to understand their growth patterns and overall health. Over 12 weeks, fish were collected from local fishermen and analyzed for their size, weight, and condition. Throughout the study period, the number of fish species varied slightly across the lakes, with Ero having the highest number. Most fish in Ero Lake showed a growth pattern indicating good environmental conditions, while fish from the other lakes showed mixed growth trends. The overall health of the fish, measured by their condition, ranged from poor to excellent, with Ero Lake generally having fish in better condition. However, no clear link was found between water quality and fish growth or condition. These findings provide valuable insights for conservation and sustainable management of fish populations in these lakes, helping to ensure their long-term health and availability for local communities.

## 1. Introduction

Freshwater fisheries ecology encompasses the intricate interactions among fish species, their aquatic habitats, and human activities [1]. Assessing the condition of aquatic ecosystems remains a significant challenge, necessitating the development of simple yet effective evaluation methods [2]. In recent years, fish have gained recognition as reliable ecological indicators of freshwater environments. Their sensitivity to environmental fluctuations, occupation of diverse trophic levels, and ease of sampling make them particularly valuable for monitoring water quality and overall ecosystem health [3].

In the context of increasing global concerns about climate change, freshwater fisheries are especially vital. They contribute significantly to biodiversity conservation and food security by supplying essential protein to millions of people, particularly in undernourished and economically disadvantaged communities [3]. In Nigeria, Ekiti State, located in the southwest, features prominently in this context. It hosts three major artificial lakes: Ureje, Ero, and Egbe. These lakes, originally developed for irrigation and potable water supply, now also support fish farming activities that offer affordable nutrition to riparian communities and the state’s population of over two million. Additionally, fisheries in these lakes create employment, sustain livelihoods, and generate revenue [4].

However, these multifunctional water bodies face growing pressure from agricultural runoff and domestic waste, which could potentially alter water chemistry and, in turn, affect the fish fauna. This possibility underscores the need for regular monitoring and ecological assessment. One of the fundamental tools for such assessments is the analysis of length–weight relationships (LWRs) in fish species [5,6]. The LWR is instrumental in fisheries science not only for estimating biomass and growth patterns but also for evaluating the ecological status of water bodies [2]. When combined with age data, the LWR offers insights into crucial biological parameters such as stock structure, size at first maturity, mortality rates, life expectancy, and reproductive strategies [6,7].

Beyond population dynamics, the LWR also supports the estimation of condition factors, which are indicators of a fish’s health and general well-being in its environment [6,8,9,10]. These indices are affected by multiple variables, including stress, sex, seasonal changes, food availability, and water quality [11,12,13]. The condition factor is particularly valuable for understanding the life history traits of fish species and for evaluating how environmental conditions influence fish physiology and survival [13,14].

Despite the abundance of inland freshwater bodies in Nigeria, up-to-date information on fish growth and health—particularly within artificial reservoirs—remains limited. This lack of data has often resulted in suboptimal management decisions, potentially hindering the development of artisanal fisheries and the sustainable use of these aquatic ecosystems.

The three study lakes (Ureje, Ero, and Egbe) experience pronounced seasonal variability due to the region’s tropical climate, which is characterized by distinct wet and dry seasons [4]. These seasonal shifts affect hydrological and ecological parameters, including water levels, nutrient input, and sedimentation. During the wet season, increased rainfall raises water levels and introduces nutrients and sediments through runoff. In response, the Ekiti State Water Corporation manages dam operations to mitigate flooding. In the dry season, the focus shifts to conserving water for irrigation, household consumption, and ecosystem stability. Routine inspections and dam safety protocols are implemented to ensure structural integrity and community safety.

This study seeks to address the data gap concerning fish population growth and health in these three artificial lakes by analyzing length–weight relationships and condition factors across various fish species. It aims to uncover spatial and temporal variations in growth and well-being, contributing to a more nuanced understanding of ecological dynamics. Ultimately, these insights can inform the development of effective management practices to sustain the ecological integrity of the lakes and support the communities that rely on them.

## 2. Materials and Methods

### 2.1. Study Site

**Figure 1:**
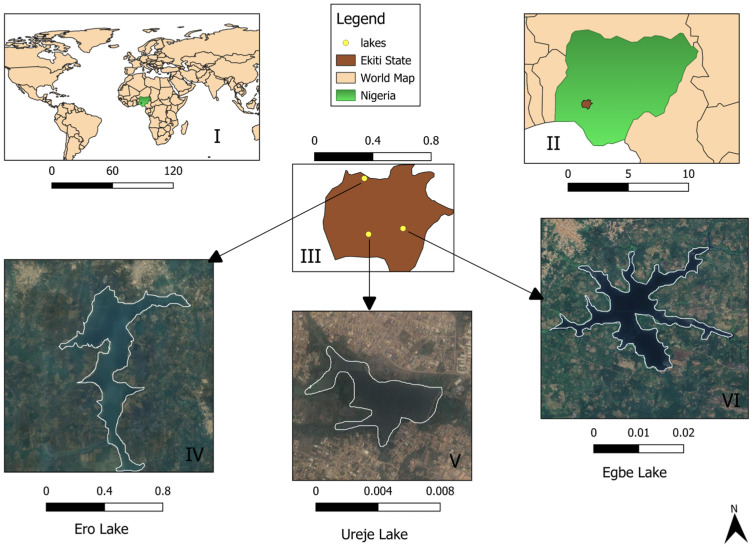
Map of the study sites, created with QGIS, showing the following: position of Nigeria on the world map (**I**); position of Ekiti State in Nigeria (**II**); position of the three lakes within Ekiti State (**III**); and Ero Lake (**IV**), Ureje Lake (**V**), and Egbe Lake (**VI**). All measurements are given in map units.

The study was conducted at three artificial lakes located in Ekiti State, Nigeria. Nigeria, a West African nation, lies between the Sahel region in the north and the Gulf of Guinea to the south, bordering the Atlantic Ocean [15]. Ekiti State is positioned between latitudes 7°15′ and 8°5′ north and longitudes 4°45′ to 5°45′ east (Figure 1). As part of the rainforest zone, Ekiti State experiences a tropical climate with distinct dry (November to March) and rainy (April to October) seasons, though recent years have seen fluctuations and a declining trend in rainfall [16].

The three lakes investigated in this study include Egbe Lake in Egbe-Ekiti (latitude 7°37′46″ N, longitude 5°33′54″ E), Ero Lake in Ikun-Ekiti (latitude 7°58′56″ N, longitude 5°11′40″ E), and Ureje Lake in Ado-Ekiti (latitude 7°35′58″ N, longitude 5°12′47″ E).

These lakes are artificial, created through the construction of dams across the Ureje, Egbe, and Ero Rivers as part of government initiatives to enhance water infrastructure in 1958, 1989, and 1985, respectively. Although these lakes are ecologically significant, with different morphometric characteristics, they have received relatively little attention in limnological research, leaving gaps in our understanding of their geomorphological characteristics.

Ero Lake possesses the largest surface area and storage volume among the three lakes (Table 1), highlighting its extensive spatial coverage. In contrast, Ureje Lake, despite being the smallest in area, records the highest mean depth, pointing to a steeper and more compact basin morphology. Egbe Reservoir, with a moderate surface area, exhibits the shallowest mean depth (Table 1), characteristic of a broader and more gradually sloping basin. Variations in maximum depth further reflect differences in vertical habitat complexity.

### 2.2. Sample Collection

Prior authorization for the study was obtained from the Ekiti State Water Corporation and the Ministry of Agriculture, through the Department of Fisheries Services. To evaluate the length–weight relationship and associated biometric indices, fish samples were collected weekly over a 12-week period, from July to October 2024 (corresponding with the peak of the rainy season). Sampling was conducted at the landing sites of Egbe, Ero, and Ureje Lakes, utilizing the daily catch of artisanal fishermen. These fishermen operated small, manually paddled canoes, deploying gill nets and traditional traps each evening and retrieving them the following morning. The traps were buoyed at the surface for ease of identification and collection. Effort was standardized across all three lakes, with consistent use of the same gear types throughout the sampling period. On each sampling day, freshly caught fish were immediately transferred in ice-packed containers to the biological laboratory of the Ekiti State Water Corporation, which maintains facilities adjacent to each lake for prompt analysis.

#### Collection of Water Samples and Analysis

Physio-chemical parameters data for the surface water of the three lakes were obtained from the Ekiti State Water Corporation for this study period. Every morning, ten sampling points were identified randomly and water samples were collected during the morning hours (8:00–10:00 a.m.) at a subsurface depth of approximately 0.3 m using sterile polyethylene plastic containers by means of direct immersion. The following in situ water quality parameters were measured: temperature (degrees Celsius), pH, electrical conductivity (μS/cm), and dissolved oxygen levels (mg/L). The Jenway 451101 water quality meter was used to measure these parameters, while the biochemical oxygen demand was analyzed following standard procedures, as described by [18].

### 2.3. Identification of Fish Samples and Measurements

Blotting paper was used to remove water from the fish bodies and the fish species were identified using standard identification manuals, as provided by [19,20]. Furthermore, the standard length (SL), which runs from the tip of the head with the mouth closed to the caudal peduncle, was measured for every fish using a measuring ruler, with a precision of 0.1 centimeters (cm). The fresh weight of the fish was also measured with a precision of 0.01 g using a digital electronic weighing balance (Ohaus CS 5000 model, Produced by Ohaus, Nänikon, Switzerland).

### 2.4. Estimation of Length–Weight Relationship

The length–weight relationship for each sampled species was evaluated using the equation originally proposed by [21]

*W* = *aL*^*b*^ (1)

where

*W* = weight of the fish (g).

*L* = length of the fish (cm).

*a* = a constant that is species-specific (the intercept).

*b* = the exponent (the slope), which indicates the type of growth (isometric or allometric).

When the value of *b* is less than 3.0, the fish is said to have experienced a negative allometric growth, but when the value of *b* is more than 3.0, the fish growth has followed the positive allometric [22]. However, when the value of *b* is equal to 3.0, the fish is said to have experienced an isometric growth [22].

The logarithmic transformation of this equation linearizes it, making it easier to analyze using linear regression. The log-transformed formula is:

*log* (*w*) = *log* (*a*) + *b log* (*L*) (2)

### 2.5. Estimation of Fish Condition Factor

The condition factor (K) [23] in fish is an index used to evaluate the overall health and well-being of individual fish or fish populations as it indicates how “fat” or “well-nourished” a fish is in relation to its length. The formula for the condition factor is:

*K* = *W*/*L*^3^ × 100 (3)

where

*K* = condition factor.

*W* = weight of the fish (g).

*L* = length of the fish (cm).

### 2.6. Statistical Analysis

Data collected during the study period were organized into separate Excel workbooks corresponding to each study site. Preliminary exploration of the dataset was performed using RStudio (version 2024.12.1, Build 563), utilizing packages within RStudio such as ggplot2, magick, factoextra vegan, and dplyr for visualization, ecological analysis, and data handling. To assess differences in the growth exponent (“b”) among fish species across the three lakes, Analysis of Covariance (ANCOVA) was applied at a 5% significance level.

The condition factor (K) was compared using Analysis of Variance (ANOVA), but only for species that occurred in all three study sites, allowing for consistent inter-site comparison. For broader comparisons of condition factors among species across the lakes, the Kruskal–Wallis test was employed to detect significant variation. To compare variations in the range of condition factors among fish species within the same lake, the coefficient of variation (standard deviation divided by the mean, multiplied by 100) was used.

To evaluate how species respond to environmental variables, Detrended Correspondence Analysis (DCA) was first conducted. The gradient length of the first axis was 1.883 (i.e., <3), indicating a linear response. Consequently, Redundancy Analysis (RDA) was used to examine potential associations between growth exponent (b), condition factor (K), and environmental variables. ANOVA was subsequently used within the RDA framework to identify which environmental factors significantly influenced both metrics.

## 3. Results

### 3.1. Water Quality Parameters

Table 2 shows that water quality parameters vary across the three lakes: Ureje Lake has the highest temperature and total dissolved solids, Ero Lake has the highest dissolved oxygen and lowest TDS and BOD, while Egbe Lake records the highest conductivity. All lakes have comparable pH levels, with Ureje showing a slightly higher mean.

### 3.2. Length–Weight Relationship

As in Table 3, the number of individuals and the species composition of samples varied across the three artificial lakes. Ero Lake had 945 individuals, which were made up of 9 species, Egbe Lake had 733 individuals representing 8 species, and Ureje Lake had 811 individuals from 7 species. Of the total 12 species encountered across the three artificial lakes, 6 of them occurred in all the three artificial lakes.

The six recurring species are *O. niloticus* (Linnaeus, 1758), *T. zilli* (Gervais, 1848), *C. guineensis* (Günther, 1862), *H. odoe* (Bloch, 1794), *C. gariepinus* (Burchell, 1822)*,* and *P. obscura* (Günther, 1862) from four families, namely, Cichlidae, Hepsetidae, Clariidae, and Channidae (Table 3).

As reported in Table 4 for Ureje Lake, *T. zilli* among the cichlids had the highest mean weight (281.17 g), while *P. obscura* had the mean size (37.35 cm). The carnivorous species (*C. gariepinus*, *P. obscura*, and *H. odoe*) showed broader size and weight ranges, with *C. gariepinus* having the highest mean weight (309.25 g). The *b* values, which reflect growth patterns, ranged from 2.42 in *T. zilli* to 2.83 in *H. odoe*, indicating predominantly allometric growth across the species.

In Egbe Lake (Table 5), the length–weight relationship parameters (*a* and *b*) vary across species, with *P. obscura* having the lowest *a* value (0.0048) and the highest *b* value (3.1758), while *T. zilli* shows the highest *a* value (0.04289) and the lowest *b* value (2.7345). Size ranges span from 12.0–22.5 cm for *C. guineensis* to 23.6–39.7 cm for *H. odoe*, with mean standard lengths ranging from 15.54 cm in *C. guineensis* to 27.63 cm in *P. obscura*. Weight ranges also show significant variation, from 39.7 g in *H. odoe* to 391.6 g in *P. obscura*, which also has the highest mean weight (190.41 ± 85.2 g), while *C. guineensis* has the lowest mean weight (108.88 ± 49.2 g).

Table 6 gives the growth report for Ero Lake where the *a* values ranged from 0.0056 for *H. niloticus* to 0.0300 for *T. zilli*, while *b* values varied between 2.84 for *C. nigrodigitatus* and 3.17 for *P. obscura*. The species with the widest size ranges were *H. niloticus* (32.1–64.9 cm) and *C. gariepinus* (26.2–55.9 cm), with mean standard lengths varying from 21.07 cm in *C. guineensis* to 38.85 cm in *H. niloticus*. Weight ranges were similarly diverse, with *H. niloticus* having the largest range (316.8–2900.0 g) and highest mean weight (633.50 ± 474.9 g), while *C. guineensis* had the smallest weight range (78.3–569.3 g) and mean weight (184.64 ± 55.6 g). The growth patterns, as indicated by *b* values, suggest predominantly positive allometric growth, with species such as *H. niloticus* and *P. obscura* showing stronger increases in weight relative to length.

### 3.3. Comparison of Length–Weight Relationship Across Artificial Lakes

To investigate spatio-temporal variation in growth patterns, the growth coefficient (b value) of these six species were compared.

(a) *Oreochromis niloticus*

**Figure 2:**
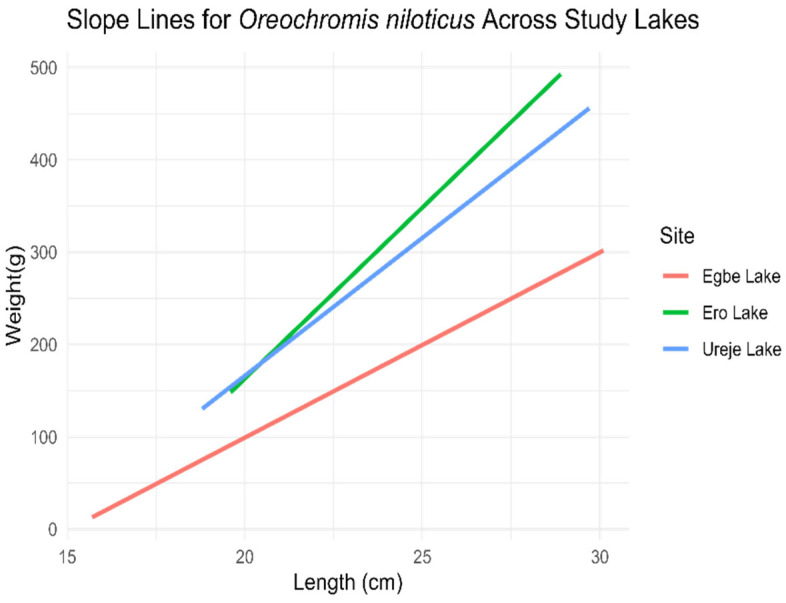
Length–weight relationship slope of *O. niloticus* across the three study sites (*log* (*w*) = *log* (*a*) + *b* *log* (*L*)).

The ANCOVA results revealed significant differences (*p* < 0.05) in the slopes of the length–weight relationship for *O. niloticus* across the three sites (Figure 2). Specifically, the slope at Egbe Lake was significantly lower than that at Ureje, indicating that *O. niloticus* in Egbe gained weight less efficiently as they increased in length. In contrast, the slope at Ero Lake was significantly higher than that at Ureje, suggesting that *O. niloticus* in Ero Lake gained weight more efficiently with increasing length.

(b) *Tilapia zilli*

**Figure 3:**
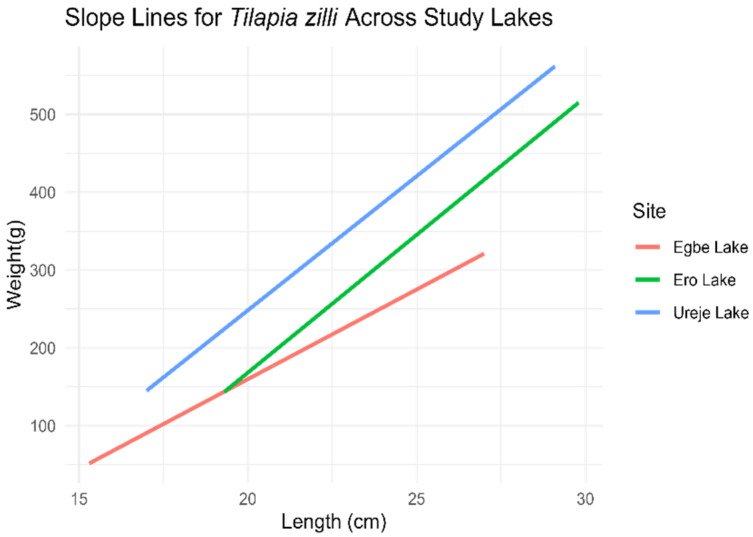
Length–weight relationship slope of *T. zilli* across the three study sites (*log* (*w*) = *log* (*a*) + *b* *log* (*L*)).

For *T. zilli*, the ANCOVA results showed significant differences (*p* < 0.05) in the slopes of the length–weight relationship across the sites (Figure 3). Fish from Egbe Lake had a significantly lower slope compared to those from Ureje Lake, indicating that *T. zilli* in Egbe Lake gained weight less efficiently as they increased in length. In contrast, the slope at Ero Lake was similar to that at Ureje Lake, suggesting that the relationship between length and weight in *T. zilli* at Ero Lake was comparable to that in Ureje Lake.

(c) *Coptodon guineensis*

**Figure 4:**
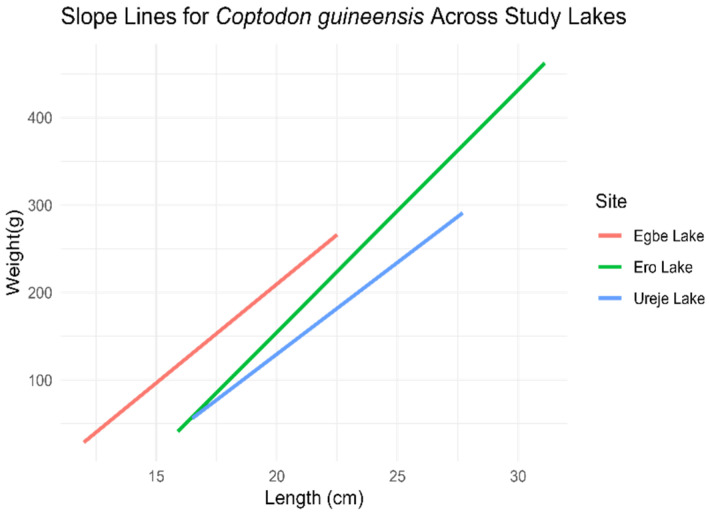
Length–weight relationship slope of *C. guineensis* across the three study sites (*log* (*w*) = *log* (*a*) + *b* *log* (*L*)).

The ANCOVA results for *C. guineensis* across the three study sites indicated that all “b” coefficients were statistically significant (*p* < 0.05). At Ureje Lake, weight increased by 20.96 units for each unit increase in length. At Egbe Lake, individuals gained weight more efficiently, with an average increase of 46.97 units per unit length increase. In contrast, fish at Ero Lake had a significantly lower average weight (109.98 units less than at Ureje Lake), but they demonstrated higher weight gain efficiency, with an increase of 6.76 units per unit increase in length. Overall, fish at Ero Lake exhibited the highest efficiency in weight gain with increasing length (Figure 4).

(d) *Clarias gariepinus*

**Figure 5:**
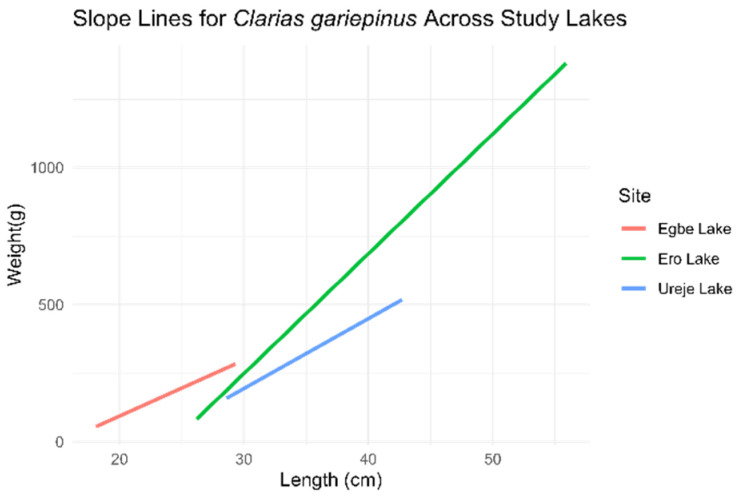
Length–weight relationship slope of *Clarias gariepinus* across the three study sites (*log* (*w*) = *log* (*a*) + *b* *log* (*L*)).

The ANCOVA results revealed statistically significant differences in all “b” coefficients (*p* < 0.05) across the three sites (Figure 5). At Ureje Lake, fish weight increased by 25.49 units for each unit increase in length. At Egbe Lake, fish showed a significant increase in average weight, with 255.96 more units compared to Ureje Lake, while Ero Lake had a significantly lower average weight, 491.98 units less than Ureje Lake. The interaction terms indicated that the slope for Egbe Lake was slightly less efficient (−5.08), while fish at Ero Lake gained weight more efficiently (+18.22) with increasing length.

(e) *Hepsetus odoe*

**Figure 6:**
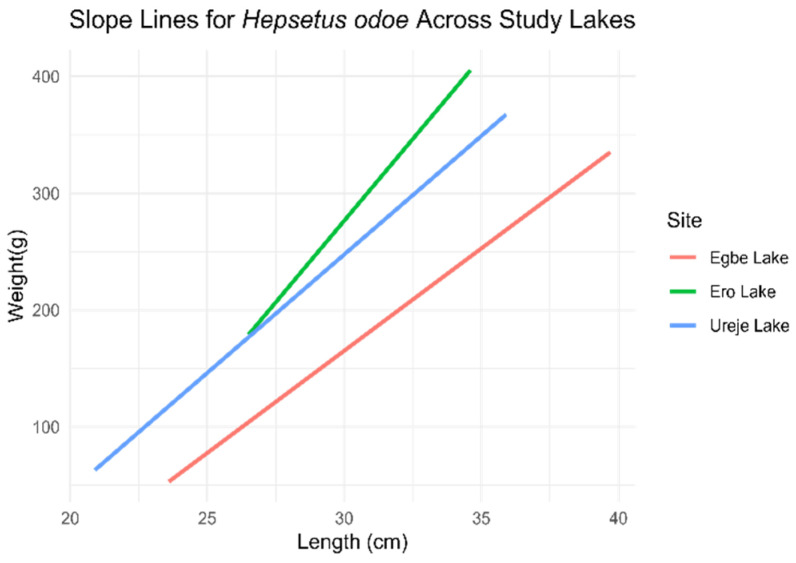
Length–weight relationship slope of *Hepsetus odoe* across the three study sites (*log* (*w*) = *log* (*a*) + *b* *log* (*L*)).

The ANCOVA revealed that most “b” coefficients were statistically significant (*p* < 0.05), except for those for individuals from Egbe Lake, which were not statistically different (*p* > 0.05) from those for individuals in Ureje Lake (Figure 6). The model explained approximately 94.62% of the variance in weight, as indicated by a multiple R-squared value of 0.9462 and an adjusted R-squared value of 0.9449, which accounted for the number of predictors. Furthermore, the F-statistic of 707.4, with a p value less than 2.2 × 10^−16^, confirming that the model was statistically significant overall.

(f) *Parachanna obscura*

**Figure 7:**
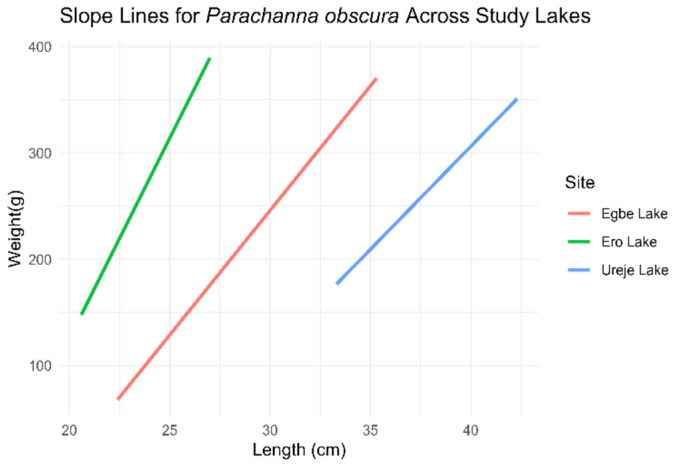
Length–weight relationship slope of *Parachanna obscura* across the three study sites (*log* (*w*) = *log* (*a*) + *b* *log* (*L*)).

The ANCOVA results for the “b” value of *Parachanna obscura* showed that, at Ureje Lake, weight increased by 19.38 units for every unit increase in length. Fish from Egbe Lake had no significant difference in average weight compared to those from Ureje. However, the interaction terms indicated that fish from both Egbe and Ero Lakes exhibited significantly steeper slopes, suggesting higher weight gain efficiency with increasing length relative to those from Ureje Lake (Figure 7).

### 3.4. Correlation Between Growth Coefficient (b Value) and Water Quality Variables

**Figure 8:**
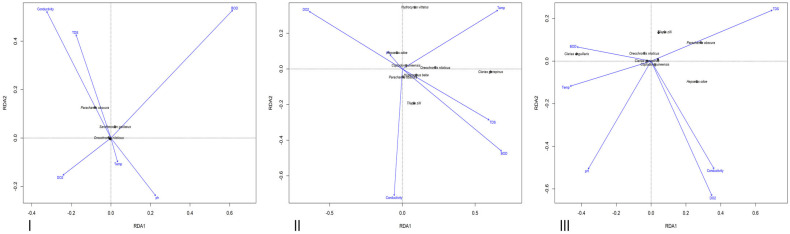
Redundancy Analysis (RDA) plots illustrating how the growth exponent (b) of each species is associated with the measured environmental variables in Ureje (**I**), Egbe (**II**), and Ero (**III**) Lakes.

The ANOVA results from the Redundancy Analysis (RDA), which examined the influence of environmental variables on the growth exponent (b), indicated that, across all three lakes, the effect of these variables was statistically insignificant (*p* > 0.05) (Table A1, Table A2 and Table A3) as seen in Figure 8.

### 3.5. Condition Factor (K)

In Ureje Lake, the condition factor of the fish species exhibited varying degrees of stability, as indicated by their respective coefficients of variation (Table 7). *T. zilli* showed the highest variability, suggesting less uniformity in individual condition, whereas *C. gariepinus* demonstrated the lowest variation, reflecting a more consistent physiological status across its population.

The coefficient of variation (CV) values for the fish species in Egbe Lake showed that *H. odoe* had the lowest variability in terms of condition factor, followed by *H. vittatus*, *C. gariepinus*, and *C. guineensis* (Table 8). The highest variability was recorded in *H. bebe*, while *O. niloticus*, *T. zillii*, and *P. obscura* exhibited moderate variation.

In Ero Lake, evaluation of the coefficient of variation (CV) revealed that *O. niloticus* displayed the highest degree of uniformity in condition factor, closely followed by *H. odoe*, *C. gariepinus*, and *C. nigrodigitatus*, all demonstrating minimal dispersion in their condition values (Table 9). *C. guineensis*, *P. obscura*, and *T. zillii* exhibited moderate levels of variability, indicating a more heterogeneous distribution within their respective condition metrics. In contrast, *C. anguillaris* and *H. niloticus* recorded the greatest spread, signifying elevated intra-species variation in condition factor.

When the K values of the six species found in all three lakes were compared using the Kruskal–Wallis test, there were no statistically significant differences (*p* > 0.05), which implied negligible variation in the condition factors across the lakes.

### 3.6. Correlation Between Condition Factor and Water Quality Variables

**Figure 9:**
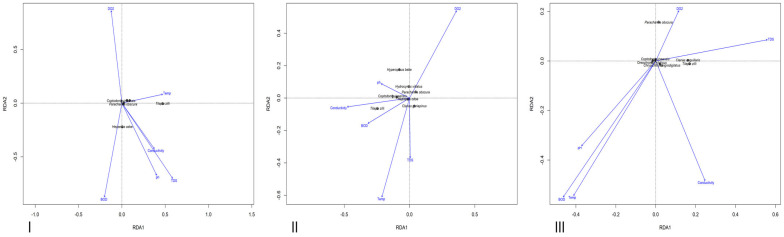
Redundancy Analysis (RDA) plots illustrating how the condition factor (K) of each species is associated with the measured environmental variables in Ureje (**I**), Egbe (**II**), and Ero (**III**) Lakes.

The ANOVA performed on the Redundancy Analysis (RDA) assessing the influence of environmental variables on condition factor (K) indicated that none of the variables had a statistically significant effect (*p* > 0.05) as seen in Figure 9, except for conductivity in Ero Lake, which showed a significant association (*p* < 0.05) (Table A4, Table A5 and Table A6).

## 4. Discussion

Length–weight relationships (LWRs) are important tools for understanding growth patterns in fish and offer insights into ecological conditions within aquatic environments. In the present study, the majority of species across the three lakes exhibited allometric growth, with estimated growth exponents (b values) deviating significantly from the isometric value of 3.0. A b value less than 3.0 indicates negative allometric growth, where fish gain weight more slowly than they increase in length, whereas values greater than 3.0 denote positive allometric growth, where weight gain exceeds linear growth [22].

Fish species in Ero Lake predominantly exhibited positive allometric growth, suggesting favorable conditions for weight gain relative to length. In contrast, species from Ureje Lake recorded the lowest b values, indicating a tendency toward negative allometric growth. These results may reflect differences in ecological productivity or stress levels among the lakes. A similar range of b values (2.78–3.54) has been reported for economically important freshwater species in the Nwaniba River, Southeast Nigeria [24], suggesting that the observed values in this study fall within expected biological limits.

Several ecological and anthropogenic factors may underline the spatial variation in growth patterns. In Ureje Lake, lower b values in species such as *H. odoe*, *O. niloticus*, and *T. zilli* may be associated with high intra-specific competition for food, particularly among similarly sized individuals (Table 1 and Table 3) [25,26]. The low “b” values observed in Egbe Lake may be linked to the impact of overfishing, which is also reflected in the smaller average size of the fish caught at this site. Previous research by [27] identified overfishing, compounded by the absence of mesh size regulations, as a major threat to the lake’s productivity. This is particularly concerning given that 57 fisherfolk operate in the area, fishing for 27 days each month. This intense fishing pressure mirrors patterns observed in other regions, such as the Nyanza Gulf of Lake Victoria, where *O. niloticus* prioritizes reproductive energy over somatic growth to adapt to stressful conditions, including fishing pressure [28]. Similarly, the impact of overfishing on growth dynamics has been documented elsewhere, with a 45% decline in average fish size and a notable reduction in growth rates along the U.S. Pacific coast over a span of 21 years [29]. Another factor which could have contributed to the difference in growth exponents (b) of fish species could be the difference in the geomorphological characteristics of these lakes [30,31]. Variations in water depth were found to have significantly influenced the growth of *O. niloticus* [30]. This may help explain the more favorable growth observed in Ero Lake.

Furthermore, seasonality likely also played a role in influencing growth exponents of the species examined. Since this study’s data were only collected during the rainy season, the results may not capture the full spectrum of growth variability. Growth coefficients can fluctuate seasonally due to changes in reproductive cycles, food availability, and metabolic demands. Previous works in Anambra River, Nigeria, reported seasonal variations in the growth exponent of six species from the family Cichlidae, including *O. niloticus* and *T. zilli* [32].

The differences in growth rates among fish species in each lake may also be attributed to their feeding habits, reflecting the unique ecological conditions of each lake. Specifically, in Ureje and Egbe Lakes, carnivorous species (*H. odoe*, *C. gariepinus*, *H. vittatus,* and *P. obscura*) exhibited faster growth rates compared to herbivorous counterparts such as *T. zilli* and *S. galilaeus*. This finding suggests that trophic level and feeding strategy influenced growth dynamics in these two lakes. This interpretation aligns with the report of [31], who found that variations in feeding habits and the availability of food sources were responsible for the differences observed in the growth coefficient (b) between 402 fish species that were studied in China.

The condition factor (K), a commonly used index of fish well-being, also provided useful insights. Although species from Ero Lake generally exhibited higher condition factor (K) values, statistical comparisons limited to species common to all three study lakes revealed no significant differences (*p* > 0.05) in K values among populations across the sites. This implies that observed differences in K may reflect individual variation rather than population-level trends. Notably, high variability in K values within certain populations (e.g., *T. zilli* in Ureje Lake) suggests differences in physiological state, potentially related to age, sex, or reproductive stage [33]. Studies have shown that during the peak spawning period, both condition factors and hepatosomatic indices decline, indicating the utilization of stored energy reserves to support reproduction [6,34].

Generally, the range of condition factors reported in this study were consistent with values reported for freshwater fish species in similar ecosystems. These includes *C. gariepinus* (0.54–1.94), *Ethmalosa fimbrata* (0.946), *Ilishia africana* (0.917), *Sardinella maderensis* (0.947), *T. zilli* (2.07), *Elops senegalensis* (0.941), *C. nigrodigitatus* (0.59–0.72), *H. vittatus* (0.47–0.74), *Lates niloticus* (0.72–0.99), and *O. niloticus* (1.32–1.61) [35,36,37,38].

Environmental parameters such as temperature, nutrient load, and pH are widely recognized as influential drivers of fish condition and growth [39,40]. However, in this study, statistical analyses revealed non-significant associations between these environmental variables and the growth exponent (b) across the three lakes. Interestingly, while agricultural runoff in rural-farming areas like those around Egbe and Ero Lakes might be expected to influence fish growth through water quality, the observed patterns suggest a more intricate dynamic, potentially shaped by a broader range of interacting factors.

Only conductivity in Ero Lake showed a significant relationship with condition factor, while other variables, including total dissolved solids, temperature, and pH, showed minimal explanatory power. This finding echoes patterns observed in some freshwater ecosystems, including temperate lakes [41], tropical reservoirs [42], and subtropical rivers [43], where growth-related indices often exhibit weak or non-linear responses to environmental gradients. Such outcomes imply that fish condition may be shaped by a combination of unmeasured factors, including species-specific physiological tolerances, behavioral adaptations, trophic interactions, and even genetic differences between populations, which can obscure or buffer the direct influence of environmental variability.

## 5. Conclusions

This study explored the health of fish populations in three lakes in Ekiti State, Nigeria (Ureje, Egbe, and Ero) by focusing on two key metrics: the length–weight relationship and the condition factor. These metrics gave valuable insights into species-specific growth patterns and overall physiological condition. While the study found significant variation in growth exponents across lakes, no strong correlations were observed between growth parameters and the measured environmental parameters. These findings highlight the need for further research into other influencing factors such as sex, reproductive stage, feeding ecology, and seasonal variation, which may play a more prominent role in shaping fish health and growth in these systems.

This research underscores the value of using simple but powerful tools like the length–weight relationship and condition factor to understand the health of aquatic ecosystems. These insights are essential for managing fisheries in a way that keeps them sustainable and productive, while also protecting the delicate balance of these ecosystems. From this study, it is evident that management actions need to be taken to manage the fisheries in these artificial lakes, considering the demand placed on them. With growing pressure on fish as a source of food, studies like this help us find smarter ways to care for both the fish and their environments, ensuring that they remain resilient for future generations.

## Figures and Tables

**Table 1:** Morphometric Characteristics of Ureje, Egbe, and Ero Lakes in Nigeria.

	Ureje Lake	Egbe Lake	Ero Lake
Year of Creation	1958	1989	1985
Surface area (Hectares)	236	590	1180
Volume (m^3^)	25 million	41 million	112 million
Maximum depth (m)	13	18	38
Mean depth (m)	10.59	6.95	9.49

Source: [4,17].

**Table 2:** Range of water quality parameters (surface water at 0.3 m depth) across the three study sites during the study period.

	Ureje Lake		Egbe Lake		Ero Lake	
	Range (min–max)	Mean ± S.D.	Range (min–max)	Mean ± S.D.	Range (min–max)	Mean ± S.D.
pH	7.3–7.8	7.54 ± 0.15	6.3–7.2	6.87 ± 0.27	6.5–7.5	7.10 ± 0.24
Temperature (°C)	23–26.8	25.01 ± 1.26	23–25.3	24 ± 0.71	22.3–25.7	23.95 ± 0.87
DO (mg/L)	4.9–6.7	5.67 ± 0.49	6.3–7.3	6.97 ± 0.28	6.9–8.2	7.39 ± 0.41
BOD (mg/L)	5.4–7.1	6.19 ± 0.59	4.1–5.7	4.76 ± 0.48	4–5.5	4.98 ± 0.46
Conductivity (μS/cm)	188.7–268.7	225.3 ± 23.9	162.9–218	195 ± 15.91	113.8–217.3	181.7 ± 29
TDS (mg/L)	97.7–174.3	130.2 ± 23.8	68.3–110.7	84.9 ± 11.04	62.7–101	81.02 ± 12.01

Note: DO = dissolved oxygen; BOD = biochemical oxygen demand; and TDS = total dissolved solids.

**Table 3:** Fish Species Identified in Ureje, Egbe, and Ero Lakes with Their Taxonomic Classification and Feeding Habits.

S/N	Order	Family	Scientific Name	Lake Found	Feeding Habit
1	Cichliformes	Cichlidae	*Oreochromis niloticus*	Ureje, Egbe, Ero	Herbivore
2	Cichliformes	Cichlidae	*Tilapia* *zilli*	Ureje, Egbe, Ero	Herbivore
3	Cichliformes	Cichlidae	*Coptodon guineensis*	Ureje, Egbe, Ero	Herbivore
4	Cichliformes	Cichlidae	*Sarotherodon galilaeus*	Ureje	Herbivore
5	Hepsetiformes	Hepsetidae	*Hepsetus odoe*	Ureje, Egbe, Ero	Carnivore
6	Anabantiformes	Channidae	*Parachanna obscura*	Ureje, Egbe, Ero	Carnivore
7	Characiformes	Alestidae	*Hydrocynus vittatus*	Egbe	Carnivore
8	Osteoglossiformes	Mormyridae	*Hyperopisus bebe*	Egbe	Omnivore
9	Osteoglossiformes	Arapaimidae	*Heterotis niloticus*	Ero	Herbivore
10	Siluriformes	Clariidae	*Clarias gariepinus*	Ureje, Egbe, Ero	Omnivore
11	Siluriformes	Clariidae	*Clarias anguillaris*	Ero	Omnivore
12	Siluriformes	Claroteidae	*Chrysichthys nigrodigitatus*	Ero	Omnivore

**Table 4:** Length–weight relationship for fish fauna in Ureje Lake, include details about length–weight parameter estimates (a and b), regression correlation coefficient (r^2^), sample size (N), standard length (SL) range, standard length (SL) means, weight range, and weight mean.

Species	a	b	R^2^	N	SL Range (cm)	SL Mean (cm) *	Weight Range (g)	Weight Mean (g) *
*O. niloticus*	0.0627	2.6452	0.9998	227	18.8–29.7	23.11 ± 2.27	146.2–492.7	259.20 ± 68.2
*T. zilli*	0.1742	2.4211	0.9998	121	17.0–29.1	20.95 ± 2.32	167.1–611.2	281.17 ± 80.7
*C. guineensis*	0.0426	2.6802	0.9997	206	16.5–27.7	22.60 ± 1.89	78.2–312.4	184.08 ± 40.0
*S. galilaeus*	0.0710	2.5475	0.9994	97	18.7–25.5	21.78 ± 1.16	123.7–271.3	183.11 ± 25.4
*H. odoe*	0.0158	2.8331	0.8203	62	20.9–35.9	30.52 ± 2.39	182.5–423.6	258.23 ± 57.4
*C. gariepinus*	0.0138	2.8249	0.9959	61	28.6–42.7	34.51 ± 2.53	179.3–552.3	309.25 ± 65.1
*P. obscura*	0.0112	2.7682	0.9910	37	33.3–42.3	37.35 ± 2.41	182.3–356.7	255.05 ± 46.9

(*) Reported as mean ± standard deviation.

**Table 5:** Length–weight relationship of fish fauna in Egbe Lake, includes details about length–weight parameter estimates (a and b), regression correlation coefficient (r^2^), sample size (N), standard length (SL) range, standard length (SL) means, weight range, and weight mean.

Species	a	b	R^2^	N	SL Range (cm)	SL Mean (cm) *	Weight Range (g)	Weight Mean (g) *
*O. niloticus*	0.0278	2.7490	0.9903	210	15.7–30.1	23.49 ± 2.88	52.8–311.2	169.25 ± 58.4
*T. zilli*	0.04289	2.7345	0.9965	102	15.3–27.0	20.40 ± 2.45	74.1–351.7	169.05 ± 57.2
*C. guineensis*	0.0375	2.8872	0.9957	208	12.0–22.5	15.54 ± 2.14	49.3–300.1	108.88 ± 49.2
*H. odoe*	0.0066	2.9647	0.9993	55	23.6–39.7	30.75 ± 4.21	39.7–77.1	178.37 ± 74.3
*H. vittatus*	0.0133	3.0291	0.9964	33	17.5–26.3	20.90 ± 2.21	77.5–266.7	136.78 ± 47.5
*C. gariepinus*	0.0195	2.8514	0.9957	56	18.1–29.3	23.51 ± 3.07	74.9–293.5	165.36 ± 63.4
*P. obscura*	0.0048	3.1758	0.9987	45	22.4–35.3	27.63 ± 3.61	88.2–391.6	190.41 ± 85.2
*H. bebe*	0.0118	2.8597	0.9362	24	17.7–27.3	22.33 ± 2.26	44.2–154.3	86.89 ± 25.3

(*) Reported as mean ± standard deviation.

**Table 6:** Length–weight relationship of fish fauna in Ero Lake, including details about length–weight parameter estimates (a and b), regression correlation coefficient (R^2^), sample size (N), standard length (SL) range, standard length (SL) means, weight range, and weight means.

Species	a	B	R^2^	N	SL Range (cm)	SL Mean (cm) *	Weight Range (g)	Weight Mean (g) *
*O. niloticus*	0.0195	3.0406	0.9979	237	19.6–28.9	23.39 ± 1.69	166.3–541.3	288.54 ± 62.9
*T. zilli*	0.0300	2.9009	0.9976	132	19.3–29.8	22.77 ± 2.11	159.3–568.3	266.16 ± 75.5
*C. guineensis*	0.0218	2.9589	0.9998	210	15.9–31.1	21.07 ± 1.96	78.3–569.3	184.64 ± 55.6
*H. odoe*	0.0099	3.0068	0.9966	90	26.5–34.6	30.45 ± 1.94	186.9–417.2	289.04 ± 54.5
*C. anguillaris*	0.0119	2.9534	0.9581	66	28.1–35.9	31.42 ± 1.75	227.5–469.2	318.56 ± 55.2
*C. gariepinus*	0.0103	2.9872	0.9998	77	26.2–55.9	32.15 ± 4.07	176.8–1698.7	342.60 ± 185.3
*C. nigrodigitatus*	0.0138	2.8479	0.9976	49	27.1–36.6	32.17 ± 2.34	165.3–393.9	274.52 ± 57.1
*P. obscura*	0.0115	3.1705	0.9928	54	20.6–27.0	25.11 ± 1.37	167.2–398.5	318.16 ± 52.1
*H. niloticus*	0.0056	3.1517	0.9997	30	32.1–64.9	38.85 ± 6.26	316.8–2900.0	633.50 ± 474.9

(*) Reported as mean ± standard deviation.

**Table 7:** The condition factor of species in Ureje Lake.

	Species	K (Range)	K (Mean) *	CV (%)
1	*C. gariepinus*	0.7094–0.7857	0.7425 ± 0.01	1.35
2	*C. guineensis*	1.4698–1.7431	1.5734 ± 0.04	2.54
3	*H. odoe*	0.7982–2.5730	0.9106 ± 0.02	2.20
4	*O. niloticus*	1.8807–2.2085	2.0603 ± 0.07	3.40
5	*P. obscura*	0.4557–0.5181	0.4847 ± 0.01	2.06
6	*S. galilaeus*	1.6362–1.8917	1.7633 ± 0.04	2.27
7	*T. zilli*	2.4803–3.4012	3.0081 ± 0.18	5.98

(*) Reported as mean ± standard deviation. CV = coefficient of variation.

**Table 8:** The condition factor of fish species in Egbe Lake.

	Species	K (Range)	K (Mean) *	CV (%)
1	*C. gariepinus*	1.0640–1.2909	1.2203 ± 0.03	2.46
2	*C. guineensis*	2.3673–3.1609	2.7554 ± 0.07	2.54
3	*H. odoe*	0.5569–0.6067	0.5820 ± 0.01	1.72
4	*H. vittatus*	1.2899–1.4939	1.4474 ± 0.03	2.07
5	*H. bebe*	0.5547–0.7971	0.7625 ± 0.05	6.56
6	*O. niloticus*	0.9431–1.5404	1.2616 ± 0.05	3.96
7	*P. obscura*	0.7641–0.8969	0.8508 ± 0.03	3.53
8	*T. zilli*	1.6900–2.1854	1.9297 ± 0.07	3.63

(*) Reported as mean ± standard deviation. CV = coefficient of variation.

**Table 9:** The condition factor of fish in Ero Lake.

	Species	K (Range)	K (Mean) *	CV (%)
1	*C. nigrodigitatus*	0.7886–0.8397	0.8138 ± 0.01	1.23
2	*C. anguillaris*	0.9758–1.2885	1.0176 ± 0.04	3.93
3	*C. gariepinus*	0.9469–0.9978	0.9797 ± 0.01	1.02
4	*C. guineensis*	1.7508–2.0880	1.9229 ± 0.03	1.56
5	*H. odoe*	0.9676–1.0529	1.0117 ± 0.01	0.99
6	*H. niloticus*	0.9489–1.0609	0.9807 ± 0.02	2.04
7	*O. niloticus*	2.0317–2.3292	2.2199 ± 0.02	0.91
8	*P. obscura*	1.9021–2.0657	1.9895 ± 0.03	1.51
9	*T. zilli*	2.0848–2.3536	2.2033 ± 0.04	1.82

(*) Reported as mean ± standard deviation. CV = coefficient of variation.

## Data Availability

All data are reported in the paper.

## Abbreviations

The following abbreviations are used in this manuscript:

Temp	Temperature
DO2	Dissolved oxygen
BOD	Biological oxygen demand
TDS	Total dissolved solids

## Appendix A

**Table A1:** ANOVA Results from RDA Assessing the Influence of Physicochemical Variables on the Growth Exponent (b) of Fish Species in Ureje Lake.

Variable	Df	Variance	F	Pr (>F)	Significance
pH	1	0.008763	0.4665	0.668	
Temp	1	0.003820	0.2034	0.887	
DO2	1	0.006988	0.3720	0.759	
BOD	1	0.054825	2.9187	0.091	
Conductivity	1	0.061488	3.2735	0.088	
TDS	1	0.028078	1.4948	0.237	
Residual	5	0.093919			

**Table A2:** ANOVA Results from RDA Assessing the Influence of Physicochemical Variables on the Growth Exponent (b) of Fish Species in Egbe Lake.

Variable	Df	Variance	F	Pr (>F)	Significance
pH	1	0.012487	0.4664	0.762	
Temp	1	0.037480	1.4000	0.297	
DO2	1	0.029871	1.1158	0.325	
BOD	1	0.020172	0.7535	0.528	
Conductivity	1	0.017398	0.6499	0.560	
TDS	1	0.004388	0.1639	0.951	
Residual	5	0.133853			

**Table A3:** ANOVA Results from RDA Assessing the Influence of Physicochemical Variables on the Growth Exponent (b) of Fish Species in Ero Lake.

Variable	Df	Variance	F	Pr (>F)	Significance
pH	1	0.007226	0.5933	0.646	
Temp	1	0.006467	0.5310	0.707	
DO2	1	0.007393	0.6071	0.665	
BOD	1	0.002191	0.1799	0.971	
Conductivity	1	0.019756	1.6222	0.193	
TDS	1	0.004065	0.3337	0.882	
Residual	5	0.060895			

**Table A4:** ANOVA Results from RDA Assessing the Influence of Physicochemical Variables on the Condition Factor of Fish Species in Ureje Lake.

Variable	Df	Variance	F	Pr (>F)	Significance
pH	1	0.0029611	1.5743	0.229	
Temp	1	0.0025664	1.3645	0.285	
DO2	1	0.0009855	0.5239	0.634	
BOD	1	0.0035340	1.8789	0.189	
Conductivity	1	0.0007209	0.3833	0.720	
TDS	1	0.0032231	1.7136	0.216	
Residual	5	0.0094043			

**Table A5:** ANOVA Results from RDA Assessing the Influence of Physicochemical Variables on the Condition Factor of Fish Species in Egbe Lake.

Variable	Df	Variance	F	Pr (>F)	Significance
pH	1	0.00021123	0.3468	0.845	
Temp	1	0.00061635	1.0119	0.384	
DO2	1	0.00087861	1.4425	0.231	
BOD	1	0.00054882	0.9010	0.464	
Conductivity	1	0.00053108	0.8719	0.486	
TDS	1	0.00158188	2.5971	0.095	
Residual	5	0.00304552			

**Table A6:** ANOVA Results from RDA Examining the Influence of Physicochemical Variables on the Condition Factor of Fish Species in Ero Lake. ** *p* < 0.01.

Variable	Df	Variance	F	Pr (>F)	Significance
pH	1	0.00016774	1.3561	0.256	
Temp	1	0.00011554	0.9341	0.511	
DO2	1	0.00010627	0.8591	0.515	
BOD	1	0.00008446	0.6828	0.633	
Conductivity	1	0.00055999	4.5273	0.008	**
TDS	1	0.00015840	1.2806	0.285	
Residual	5	0.00061846			
